# Signal Acquisition-Independent Lossless Electrocardiogram Compression Using Adaptive Linear Prediction

**DOI:** 10.3390/ijerph20032753

**Published:** 2023-02-03

**Authors:** Krittapat Bannajak, Nipon Theera-Umpon, Sansanee Auephanwiriyakul

**Affiliations:** 1 Department of Electrical Engineering, Chiang Mai University, Chiang Mai 50200, Thailand; 2 Biomedical Engineering Institute, Chiang Mai University, Chiang Mai 50200, Thailand; 3 Department of Computer Engineering, Chiang Mai University, Chiang Mai 50200, Thailand

**Keywords:** electrocardiogram (ECG), lossless compression, linear prediction, Golomb–Rice coding

## Abstract

In this paper, we propose a lossless electrocardiogram (ECG) compression method using a prediction error-based adaptive linear prediction technique. This method combines the adaptive linear prediction, which minimizes the prediction error in the ECG signal prediction, and the modified Golomb–Rice coding, which encodes the prediction error to the binary code as the compressed data. We used the PTB Diagnostic ECG database, the European ST-T database, and the MIT-BIH Arrhythmia database for the evaluation and achieved the average compression ratios for single-lead ECG signals of 3.16, 3.75, and 3.52, respectively, despite different signal acquisition setup in each database. As the prediction order is very crucial for this particular problem, we also investigate the validity of the popular linear prediction coefficients that are generally used in ECG compression by determining the prediction coefficients from the three databases using the autocorrelation method. The findings are in agreement with the previous works in that the second-order linear prediction is suitable for the ECG compression application.

## 1. Introduction

Cardiovascular disease (CVD) has become the second major cause of death of non-communicable diseases (NCDs) for several years. Although the trend of NCD mortality was decreased over the last 20 years, the COVID-19 pandemic has aggravated that trend by increasing the risk of illness and death for the existing NCD patient [1].

An electrocardiogram (ECG) signal, consisting of the P, T, and U waves and the QRS complex, represents the electrical activity of the heart [2]. For the diagnosis and treatment of CVD, monitoring and recording of the ECG signals are required. The ECG signal can represent electrical activities, heart rate variability (HRV), and abnormalities of the heart. One approach to the ECG application is telemedicine, which is the implementation of the internet of things (IoT), wireless sensor networks, and embedded systems in the medical system. Also, multi-lead ECG acquisition, long-term monitoring and recording, higher resolution, and frequency of the signal are required for better ECG monitoring and diagnosis. To improve the performance and efficiency of the data transmission, data storage, and clinical application, the data compression process for the ECG signal is important, especially for those requirements mentioned before.

The compression method for the ECG signal is approached in two directions, i.e., lossy and lossless compression. Lossy compression significantly has a higher compression ratio than lossless compression, where the compression ratio is the ratio of the original data size to the compressed data size. However, the lossy compression generally has some reconstruction error which means the reconstructed signal value is different from the original one. While the lossless term for the ECG signal compression implies no error from the reconstructed signal.

The ECG compression method can also be categorized into three techniques, i.e., direct-time domain, transform domain, and parameter extraction [3]. The direct-time technique compresses the data in the time domain directly by extracting the significant samples and removing redundancies. The transform domain technique compresses the data by transforming the data to another domain, for example, Fourier transform, wavelet transform, discrete cosine transform, etc. The parameter extraction technique compresses the data by extracting the characteristics of features of the signal, for example, linear prediction, complex or peak extraction, neural network approach, etc. In recent years, most of the compression methods have been based on two main approaches, i.e., the linear prediction technique and the transform domain technique. In addition, the coding and data packaging techniques are also implemented to maximize the compression performance.

Tsai and Kuo [3] proposed the adaptive linear prediction with content-adaptive Golomb–Rice coding. Tsai and Tsai [4] proposed the multi-channel adaptive linear prediction with a modified Golomb–Rice coding method, and Tsai et al. [5] also implemented the method in [4] on Very Large-Scale Integration (VLSI). This work compressed the 12-lead ECG signal by using four reference leads to calculate the other eight leads of the signals. The fuzzy theory and exponential weighting technique were applied to the adaptive linear prediction. Deepu et al. [6] introduced sign-sign least mean square (SSLMS) adaptive linear prediction with the Savitzky-Golay filter. Deepu et al. [7] proposed linear prediction and fixed-length data packaging and also introduced the 1st to 4th order of the linear prediction function for the ECG signal. Arnavut [8] introduced the Burrow-Wheeler transform with the autoregressive (AR) model, the Move-to-front coder, and the Inversion ranks coder with the linear prediction compression method. Luo et al. [9] proposed the adaptive linear prediction based on a fuzzy decision with 2-stage Huffman coding implemented on VLSI. Jia et al. [10] proposed the dual-mode linear prediction compression method with context-based error modeling and modified Golomb–Rice coding. Tseng et al. [11] introduced the compression method using Takagi-Sugeno fuzzy neural network with predictive coding. Miaou and Chao [12] proposed wavelet-based vector quantization for lossless compression with a unified vector quantization framework. Koski [13] introduced an example of ECG compression with various encoding methods, including 1st and 2nd-order linear prediction, Gamma coding, Huffman coding, LZ-77 method, and complex extraction. Giuseppe et al. [14] proposed an ECG lossless compression method using the RAKE algorithm with simple prediction. Zhou [15] introduced the K-means clustering for lossless ECG compression by applying the K-means clustering with the QRS complex and the 1st-order differential for non-QRS region. Li et al. [16] proposed the differential linear prediction method with modified variable-length coding.

This paper is organized as follows. The proposed method and the related techniques, including the prediction error-based linear prediction, the modified Golomb–Rice coding, the data reconstruction process, and the linear prediction coefficient determination, are described in Section 2. Section 3 describes the 3 ECG data sets used in this research. The experimental results are presented and discussed in Section 4 and Section 5, respectively. Section 6 then concludes the paper.

## 2. Materials and Methods

**Figure 1:**
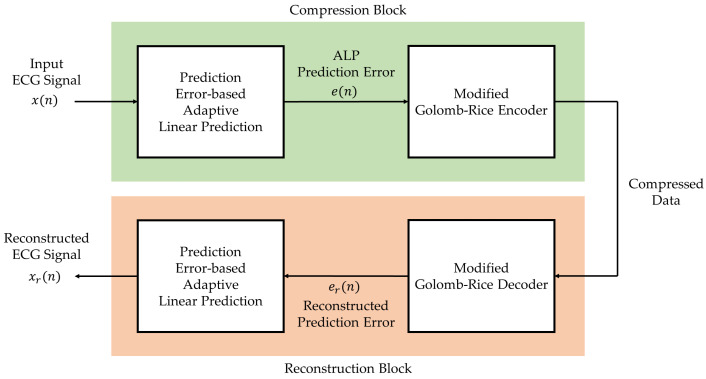
Block diagram of the proposed lossless ECG compression method.

The proposed compression method aims to increase the compression ratio, which is the ratio of the original data size to the compressed data size while maintaining the lossless feature. In addition, the compression method was designed to be operable in real time without determining any parameters or thresholds. The method proposed in this work is based on the lossless compression concept. The adaptive linear prediction was introduced in recent works where the linear prediction function was changed according to the trend of the ECG signal. However, this concept does not bring the lowest linear prediction error, especially in regions between intervals, abnormal beats, and noisy regions. In addition, the thresholds of some parameters for determining the linear prediction functions may be changed when the data sets or the acquisition setups of the ECG signals are different. The encoding process in [5] is applied in this work since the process can adaptively adjust the parameters according to the input signal. The overall scheme of the proposed ECG compression method comprising the compression stage and reconstruction stage is shown in Figure 1.

The compression stage for ECG signal in this work includes 2 processes, i.e., the prediction error-based adaptive linear prediction and the Golomb–Rice coding. The concept of this compression stage is to minimize the ECG signal level into the prediction error for each sample by using linear prediction and to encode the prediction error to the binary format by using length-variable coding. Meanwhile, the reconstruction of the compressed signal data can be done by reversing the compression processes. The related techniques are described in more detail next.

### 2.1. Prediction Error-Based Adaptive Linear Prediction

The linear prediction has been implemented in many related works for ECG compression since it has the benefit of low complexity for implementation in hardware such as Silicon-on-Chip (SoC) and Field Programmable Gate Array (FPGA). In this research, we exploited the modified forward linear prediction for the linear prediction process. The predicted value ${\hat{x}}_{p}\left(n\right)$ for the linear prediction order p of the signal x(n) can be predicted from the linear combination of the past signal value x(n−i) with the linear prediction function in Equation (1) where $a_{p}\left(i\right),\;i=1,\;p,$ are the linear prediction coefficients,

(1) $${\hat{x}}_{p}\left(n\right)=\sum_{i=1}^{p}a_{p}\left(i\right)x\left(n-i\right)$$

and the prediction error $e_{p}\left(n\right)$ of the prediction order p can be defined with Equation (2), i.e.,

(2) $$e_{p}\left(n\right)=x\left(n\right)-{\hat{x}}_{p}\left(n\right)$$

Therefore, the main factor that affects the compression ratio in this approach is the prediction error. The compression ratio can be maximized by minimizing the prediction error. This means the linear prediction order and the linear prediction coefficients must be determined or selected appropriately for each ECG signal. One of the methods to determine the linear prediction coefficients is by using the autocorrelation method, which can be solved by using the Yule–Walker equations with the Levinson–Durbin algorithm [17]. In terms of adaptive linear prediction (ALP), the order of the linear prediction function can be changed following the trends or the region of the ECG signal to give the lowest prediction error. We initially proposed a compression method and tested it on small data in [18]. The algorithm of the prediction error-based adaptive linear prediction consists of 2 steps.

The first step is to calculate the prediction value and the prediction error of each linear prediction function from the 1st to 4th order. These coefficients of the linear prediction were used in many related works using the triangle of binomial transform coefficients like Pascal’s triangle. All 4 linear prediction functions are shown in Equations (3)–(6), respectively.

(3) $${\hat{x}}_{1}\left(n\right)=x\left(n-1\right)$$

(4) $${\hat{x}}_{2}\left(n\right)=2x\left(n-1\right)-x\left(n-2\right)$$

(5) $${\hat{x}}_{3}\left(n\right)=3x\left(n-1\right)-3x\left(n-2\right)+x\left(n-3\right)$$

(6) $${\hat{x}}_{4}\left(n\right)=4x\left(n-1\right)-6x\left(n-2\right)+4x\left(n-3\right)-x\left(n-4\right)$$

The second step is to compare the absolute value of each prediction error from the first step. The smallest absolute prediction error is chosen to be the determined absolute prediction error as shown in Equation (7),

(7) $$e\left(n\right)=\underset{}{\min}\left\{\left|e_{p}\left(n\right)\right|\right\}$$

**Figure 2:**
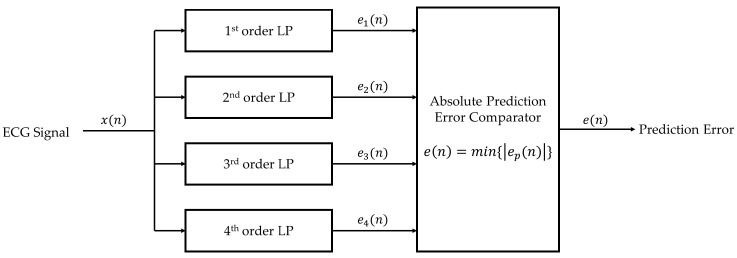
Block diagram of the prediction error-based adaptive linear prediction.

The block diagram that summarizes the prediction error-based adaptive linear prediction method is shown in Figure 2.

**Figure 3:**
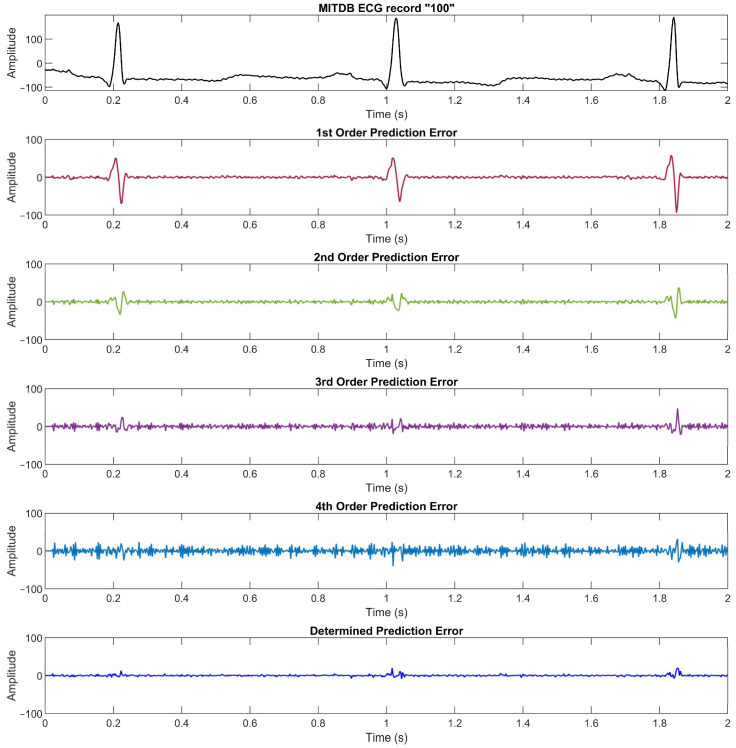
The ECG signal, prediction errors for linear prediction orders 1, 2, 3, and 4, and the determined prediction error of MITDB record number “100”. To convert the amplitude to millivolts, divide the value by 200.

Figure 3 illustrates an ECG signal from the MIT Arrhythmia database record number “100” along with its prediction errors from the 1st to 4th linear prediction orders and the determined prediction error from Equation (7). We can see that the lower order of linear prediction function yields a better prediction error in the flat region, whereas the higher order yields a better prediction error in the high slope region.

### 2.2. Modified Golomb–Rice Coding

The concept of entropy coding is to encode the data in length-variable code format. Many related works introduced Huffman coding and Golomb–Rice coding for data compression since they are lossless compression methods. Golomb–Rice coding was introduced by Solomon W. Golomb [19] and was modified by Robert F. Rice [20,21]. The idea of this method is to convert the data value to the quotient and remainder by using the power of 2, which is suitable for storing the compressed data in binary format.

The modified Golomb–Rice coding process converts the prediction error to a non-negative integer value since the prediction error from the linear prediction process is the signed integer value, while the encoding input must be a non-negative value [5]. For the non-negative value M(n), the prediction error e(n) can be converted by Equation (8).

(8) $$M\left(n\right)=\{\begin{array}{c} 2e\left(n\right)\;\;\;\;\;\;\;\;\;\;\;\;\;\;\;,e\left(n\right)\geq 0\\ 2\left|e\left(n\right)\right|-1\;\;\;\;\;\;,e\left(n\right)<0 \end{array}$$

The non-negative prediction error is then encoded into the quotient and remainder by using the power k(n) of 2 in Equations (9) and (10),

(9) $$q\left(n\right)=[M\left(n\right)/2^{k\left(n\right)}]$$

(10) $$r\left(n\right)=M\left(n\right)\;\mathrm{mod}\;2^{k\left(n\right)}$$

where k(n) is the base-2 logarithm of the mean absolute error (MAE) of the 3 latest samples, defined as Equations (11) and (12),

(11) $$\mathrm{MAE}\left(e\left(n\right)\right)=\frac{\sum_{i=1}^{3}\left|e\left(n-i\right)\right|}{3},$$

(12) $$k\left(n\right)=\log_{2}\left(\mathrm{MAE}\left(e\left(n\right)\right)\right)$$

The compressed data c(n) includes the quotient q(n), which is encoded in unary format, one of the ‘0′ bit for the quotient and the remainder separation, and the remainder r(n), which is encoded in binary format, i.e.,

(13) c(n)=[q(n),0,r(n)]

**Figure 4:**
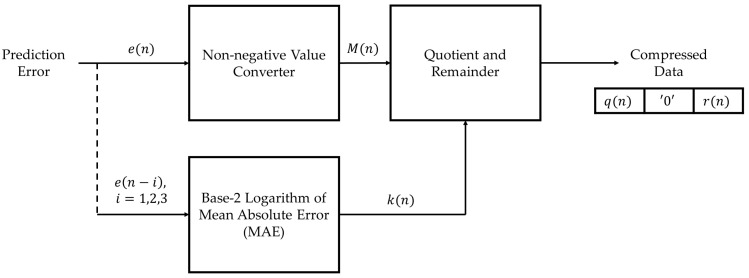
Block diagram of the modified Golomb–Rice coding.

The length of the compressed data depends upon the absolute prediction error and the parameter k(n). The block diagram of the Golomb–Rice coding can be shown in Figure 4.

**Figure 5:**
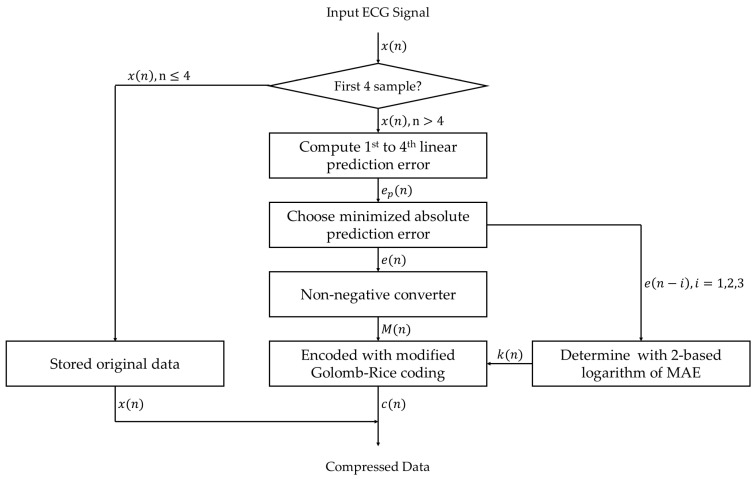
Flowchart of the proposed ECG lossless compression method.

The flowchart of the proposed ECG lossless compression method is shown in Figure 5.

### 2.3. Data Reconstruction Process

**Figure 6:**
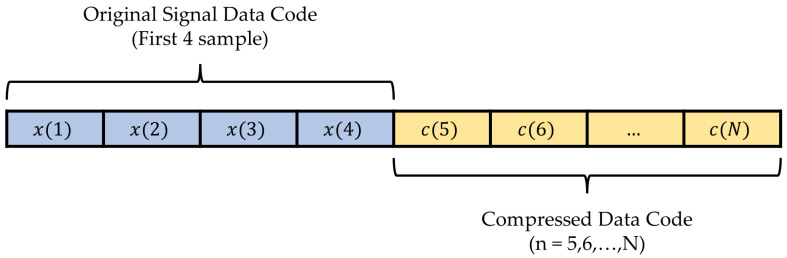
Data package of the compressed data.

The ECG signal can be reconstructed back by reversing the compression process. The compressed data is encoded with the modified Golomb–Rice coding as described earlier. Then the reconstructed ECG signal value $x_{r}\left(n\right)$ can be computed with Equation (2) since ${\hat{x}}_{p}\left(n\right)$ can be computed with the 4 recently reconstructed signals, and e(n) is received as the compressed data. The reconstructed signal $x_{r}\left(n\right)$ will be the same as the original ECG data x(n). Hence, this is a lossless compression method since there is no reconstruction error. Note that the first 4 samples of the ECG signal data must be encoded and stored directly to compute the 1st to 4th order of the linear prediction at the compression and reconstruction process. The data packet of the compressed data is shown in Figure 6.

**Figure 7:**
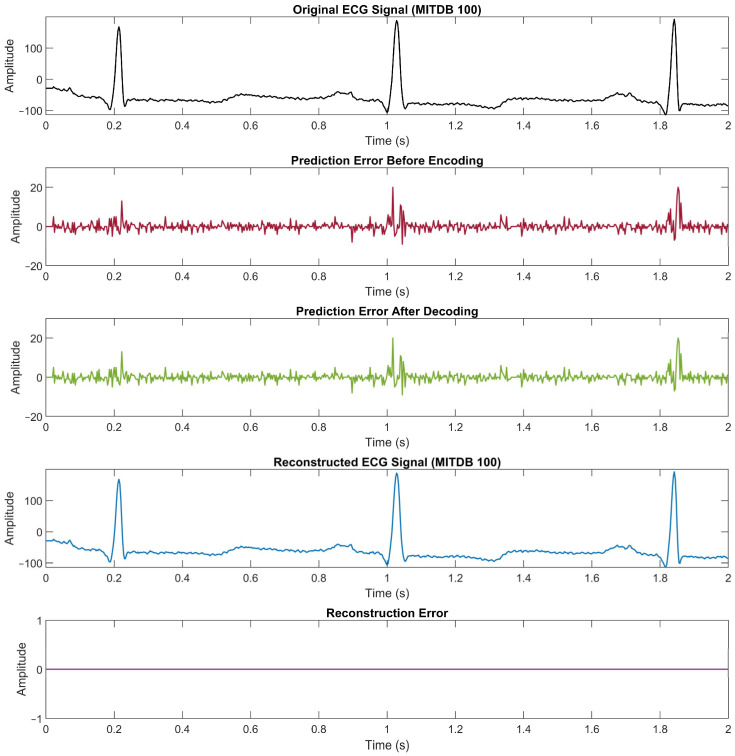
Example of signals in the proposed lossless ECG compression method: original ECG signal, prediction error before encoding and after the decoding process, reconstructed ECG signal, and the reconstruction error for the MITDB record number “100”. To convert the amplitude to millivolts, divide the value by 200.

Figure 7 illustrates the proof of the lossless compression method. The first plot is an example of ECG signal data from the record number “100” with 2 s in time duration. The second plot is the prediction error that is determined before the encoding process. Then the third plot is the decoded prediction error from the compressed data, whereas the fourth plot is the reconstructed ECG signal data. The last plot shows the difference between the original data and the reconstructed data to confirm that there is no reconstruction error in the proposed method. The compression ratio achieved from this example is 3.63.

### 2.4. Linear Prediction Coefficient Determination

As for a linear prediction-based ECG compression method, the prediction order and coefficients are very crucial. Some questions still remain to answer, for example, which of Equations (1)–(4) is the best for ECG signal prediction? Are the coefficients in those equations good enough? Hence, they deserve a close investigation of how or how well we could come up with them. Consider a function that predicts the current sample by using the linear combination of the past samples as in Equation (1); the linear prediction coefficients are a set of coefficients that minimize the prediction error

(14) $$e\left(n\right)=x\left(n\right)-\sum_{i=1}^{p}a_{p}\left(i\right)x\left(n-i\right)$$

One of the approaches to determine the coefficients $a_{k}$, k = 1, 2, …, p, is by using the autocorrelation method [17]. Yule–Walker equations were used to solve the coefficients with the autocorrelation of the signal x(n), i.e., solve the following equation:

(15) $$\left[\begin{array}{ccccc} R\left(0\right) & R\left(1\right) & R\left(2\right) & \cdots & R\left(p-1\right)\\ R\left(1\right) & R\left(0\right) & R\left(1\right) & \cdots & R\left(p-2\right)\\ R\left(2\right) & R\left(1\right) & R\left(0\right) & \cdots & R\left(p-3\right)\\ \vdots & \vdots & \vdots & \ddots & \vdots \\ R\left(p-1\right) & R\left(p-2\right) & R\left(p-3\right) & \ldots & R\left(0\right) \end{array}\right]\left[\begin{array}{c} a_{1}\\ a_{2}\\ a_{3}\\ \vdots \\ a_{p} \end{array}\right]=-\left[\begin{array}{c} R_{1}\\ R_{2}\\ R_{3}\\ \vdots \\ R_{p} \end{array}\right]$$

where R(k) is the autocorrelation of x(n) for time lag k=1,2,…,p. This equation can be solved by using the well-known Levinson–Durbin recursive procedure [22].

## 3. ECG Data Sets

For the performance evaluation for data compression in this research, we used three public data sets of ECG signals, i.e., the PTB Diagnostic ECG database (PTBDB), the European ST-T database (EDB), and the MIT-BIH Arrhythmia database (MITDB). As mentioned earlier, we initially tried to compress a part of MITDB in [18]. However, more experiments needed to be done, and some issues needed to be investigated. Hence, in this research, extensive experiments were performed on these three ECG data sets. It is worthwhile noting that these three data sets have totally different characteristics [23] as follows:

1. PTB Diagnostic ECG database (PTBDB) [24]:
  - 549 records
  - Mostly 2 min duration
  - 1000 Hz of sampling frequency
  - 16-bit resolution with 2000 A/D units per mV
2. European ST-T database (EDB) [25]:
  - 90 records
  - 120 min duration
  - 250 Hz of sampling frequency
  - 12-bit resolution with 200 A/D units per mV.
3. MIT-BIH Arrhythmia database (MITDB) [26]:
  - 48 records
  - 30 min duration
  - 360 Hz of sampling frequency
  - 11-bit resolution with a 10-mV voltage range.

As we can see, these three data sets are different in all of these features, i.e., number of records, time duration per record, sampling frequency, and resolution. Hence, they will be very good candidates to be used in the evaluation of lossless ECG compression across different data sets or different setups.

## 4. Experimental Results

### 4.1. Lossless Compression Performance

The performance of a data compression method is defined in terms of the compression ratio (CR), i.e.,

(16) $$\mathrm{CR}=B_{0}/B_{C}$$

where $B_{0}$ is the original ECG data size and $B_{C}$ is the compressed ECG data size.

The compression ratio can indicate the reduced data size of the compressed data compared to the original data size. The larger compression ratio means more data size has been reduced. The average compression ratio from all records of each database is used to compare the compression performance with other methods. For the lossy compression method, the reconstructed error of the compressed signal data is also defined by using Percent Root-Mean-Square Difference (PRD). Since there is no reconstruction error for the lossless compression, the PRD for the lossless compression is always 0.

**Figure 8:**
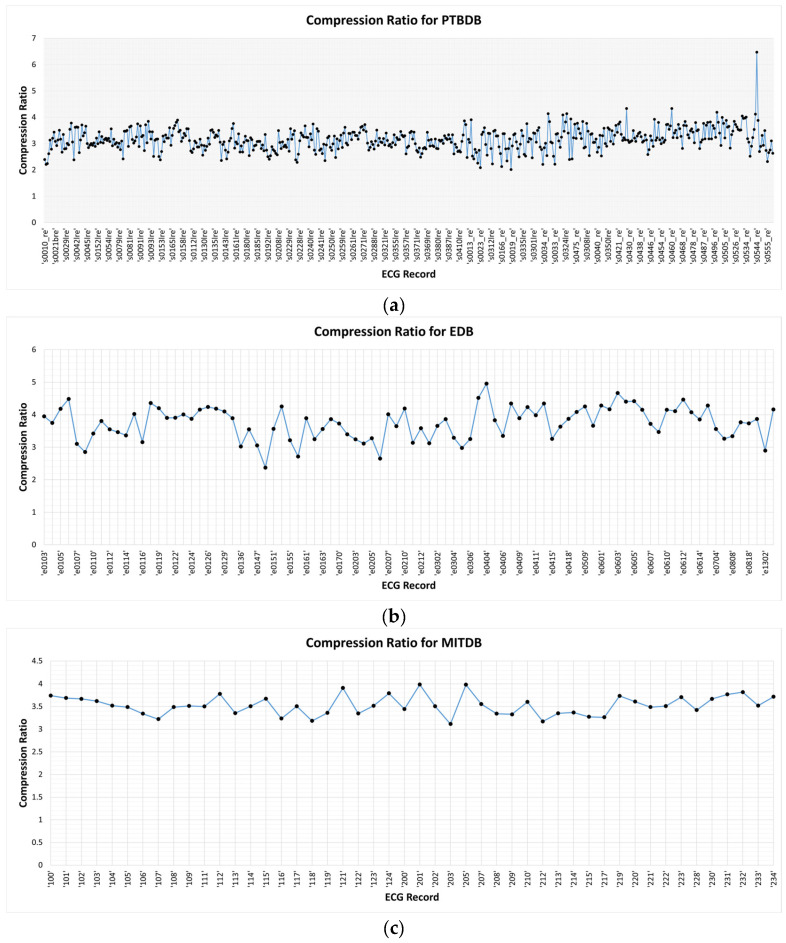
Compression ratio for each record of the data sets (**a**) PTBDB, (**b**) EDB, (**c**) MITDB.

For the PTBDB ECG records, the EDB ECG records, and the MITDB ECG records, the proposed compression method achieved average compression ratios of 3.16, 3.75, and 3.52, respectively. Figure 8a,b,c illustrates the compression ratio achieved for each record from the PTBDB, EDB, and MITDB data sets, respectively. These results clearly demonstrate different ranges and deviations of compression ratios in different data sets.

To evaluate the data compression performance of the proposed method to other state-of-the-art methods, its compression ratios achieved are compared to the related works for PTBDB, EDB, and MITDB data sets, as shown in Table 1, Table 2 and Table 3, respectively.

### 4.2. Linear Prediction Coefficient Comparison

For the compression method proposed in this research, the linear prediction coefficients are determined by using Pascal’s triangle of binomial transform coefficients, as shown in Equations (3)–(6). In the related works, each order of the linear prediction with these coefficients was used for different regions or trends of the ECG signal. The low-order linear prediction function yields a lower prediction error at the flat region, while the high-order yields better performance in the complex interval or the high slope region. In [6], it was mentioned that the 2nd order of the linear prediction function yielded the best performance from the 1st to 4th order by comparing mean absolute prediction error and root-mean-square prediction error.

In this section, the linear prediction coefficients for the ECG signal were determined by using the autocorrelation method mentioned in Section 2.4. The highest order to determine the coefficients was varied from 1 to 4, and each coefficient was also rounded to an integer value. This would make it usable in the lossless compression method since all the signal values and parameters must be in the integer format. The proposed and average determined coefficients are shown in Table 4 and Table 5, respectively. In addition, the average determined coefficients rounded to the nearest integers are shown in Table 6.

## 5. Discussion

From the compression results compared to the state-of-the-art works in Table 1, Table 2 and Table 3 in Section 4.1, the proposed compression method achieved compression ratios close to or better than that of the others. The results demonstrate that the proposed lossless ECG signal compression method is very robust. It can be applied even in different ECG signal acquisition setups, i.e., different numbers of records, different time duration per record, different sampling frequencies, or different resolutions (number of bits per sample). For the PTBDB data set, the result in [4,5] showed CR of 3.98 and 4.07, which is higher than the 3.16 achieved by the proposed method. However, we cannot really compare them because, in [4], data from other channels were required to reconstruct the data in the channel of interest. It was not a single-lead ECG signal reconstruction like what we have here. For the EDB and MITDB data sets, it is interesting that the results in [8] yielded very high CRs compared to other existing methods. In [8], however, it was mentioned that not all data were used; there was data selection to make it comparable to a previous method. Meanwhile, we applied the proposed method for the entire data in each data set. Hence, the results may not be comparable. To make a fair comparison, we need to make sure that all methods take the exact same ECG signals as the inputs.

**Figure 9:**
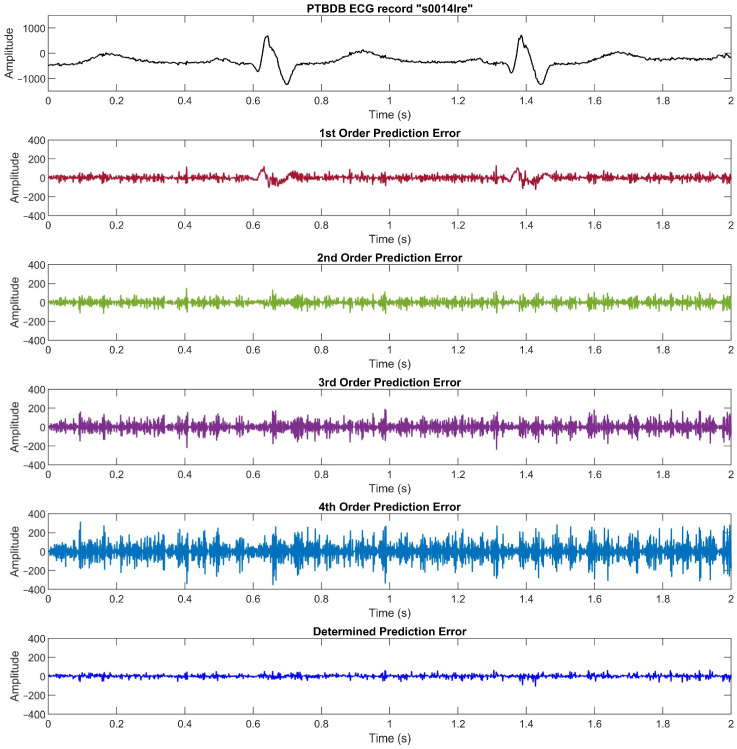
The ECG signal, prediction errors for linear prediction orders 1, 2, 3, and 4, and the determined prediction error of PTBDB record “s0014lre”. To convert the amplitude to millivolts, divide the value by 2000.

**Figure 10:**
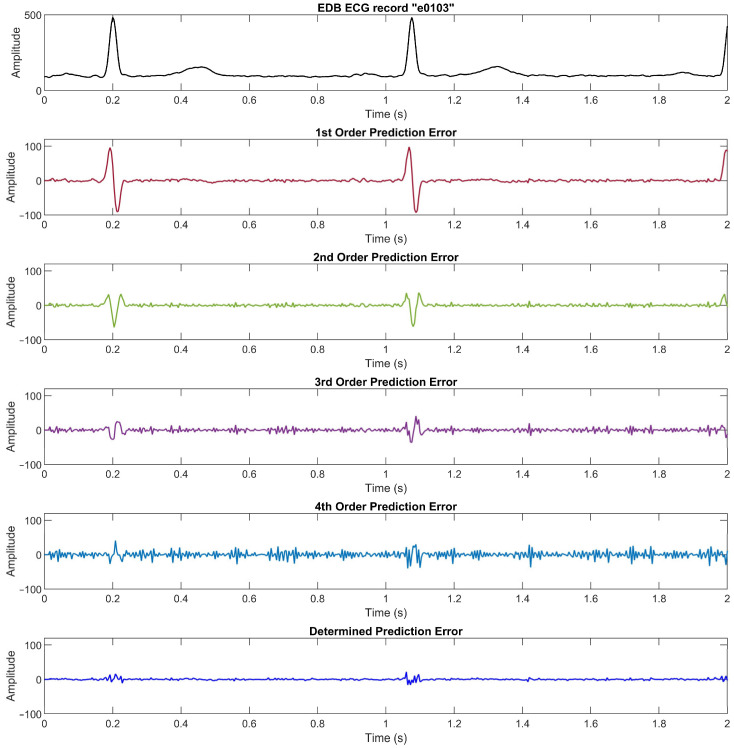
The ECG signal, prediction errors for linear prediction orders 1, 2, 3, and 4, and the determined prediction error of EDB record “e0103”. To convert the amplitude to millivolts, divide the value by 200.

We compare the prediction error for each order and the determined absolute prediction error from the MITDB data set in Figure 2 (see Section 2.1), the PTBDB data set in Figure 9, and the EDB data set in Figure 10. The results in these figures show that the higher resolution and sampling frequency of the ECG signal will cause a higher prediction error which in turn decreases the compression ratio. In the PTBDB data set, the high frequency and resolution of the original signals also significantly increases the low-order prediction errors in the flat regions, whereas it is lower for the MITDB and EDB data sets. This is the reason that the average CR for the PTBDB data set is lower than that for the MITDB and EDB data sets.

From the results shown in Table 4, Table 5 and Table 6 in Section 4.2, the proposed and algorithm-determined linear prediction coefficients were compared. Each order of the proposed coefficient was chosen to minimize the prediction error for the different signal regions. Meanwhile, the determined coefficient was calculated to fit the entire length of the signal. Therefore, the results show that the 2nd order of the linear prediction function is sufficient to predict the ECG signal with an acceptable prediction error. The corresponding coefficients are $a_{1}=2$ and $a_{2}=-1$. It is very interesting that although all three data sets are different in signal acquisition setups, especially the sampling frequency, which dictates the temporal information, they still produce the same rounded prediction coefficients. This is in agreement with the results in [7] in that, using the mean absolute prediction error and root-mean-square prediction error, the 2nd order of the linear prediction function yielded the best prediction performance.

## 6. Conclusions

In this research, a lossless ECG signal compression method was proposed. The method exploited the prediction error-based adaptive linear prediction and the modified Golomb–Rice coding. The evaluation results of the proposed method on the PTB Diagnostic database, the European ST-T database, and the MIT-BIH Arrhythmia database achieved an average compression ratio (CR) of 3.16, 3.75, and 3.52, respectively. These average CRs on these three data sets are comparable to or better than most of the other related works. The results also suggested that the proposed lossless ECG signal compression method was very robust and could be applied in different ECG signal acquisition setups, i.e., numbers of records, time duration per record, sampling frequencies, or resolution. In addition, the proposed linear prediction coefficients were compared to the coefficients determined by the autocorrelation method with Yule–Walker equations and the Levinson–Durbin method. The results demonstrated the agreement with previous work, i.e., the 2nd order of the linear prediction function is suitable to apply to ECG signal compression with the corresponding prediction coefficients $a_{1}=2$ and $a_{2}=-1$. Even though the proposed method is robust to different ECG signal acquisition setups, its obvious limitation is that it is specifically designed for ECG signals only. The ongoing and future trends of this work include how to further increase the compression ratio in this lossless ECG signal compression and how to implement the proposed method to other signals in which lossless compression is required. We can anticipate that the determination of the prediction coefficients of each particular signal will be crucial. If the proper prediction coefficients are determined, efficient lossless compression will be achieved by the proposed method.

## Figures and Tables

**Table 1:** The comparison of the average compression ratio to other methods on PTBDB data set.

Ref.	Compression Method	Average CR
[4]	Multi-channel ALP (1st, 2nd, and 4th order weight average) + Golomb–Rice Coding	3.98 (Multi-lead)
[5]	Multi-channel ALP (1st,2nd, and 3rd order weight average) + Golomb–Rice Coding	4.07 (Multi-lead)
Proposed	Prediction error-based ALP + Golomb–Rice Coding	3.16 (Single-lead)

**Table 2:** The comparison of the average compression ratio to other methods on EDB data set.

Ref.	Compression Method	Average CR
[8]	Burrow–Wheeler Inversion Coding	4.77
[12]	Wavelet-based Vector Quantization	3.499
[16]	Differential Linear Prediction + MVLC	2.45
Proposed	Prediction Error-based ALP + Golomb–Rice Coding	3.75

**Table 3:** The comparison of the average compression ratio to other methods on MITDB data set.

Ref.	Compression Method	Average CR
[3]	ALP + Context-based Golomb–Rice Coding	2.835
[4]	ALP + Golomb–Rice Coding	2.89
[6]	SSLMS ALP + Savitzky–Golay Filter	2.15
[7]	ALP + Fixed-length Coding	2.25
[8]	LP + Burrow–Wheeler Inversion Coding	4.24
[9]	Fuzzy-based ALP + 2-stage Huffman Coding	2.53
[10]	Dual-mode LP + Error Modeling + Golomb–Rice Coding	2.975–3.040
[11]	Takagi–Sugeno fuzzy NN Predictive Coding	3.22
[12]	Wavelet-based Vector Quantization	3.031
[14]	RAKE Coding Algorithm	2.67
[15]	K-means Cluster + Differential Coding	2.49 (12-bit Length)
Proposed	Prediction Error-based ALP + Golomb–Rice Coding	3.525

**Table 4:** Proposed Linear Prediction Coefficients.

Data Sets	$\mathrm{Proposed}\;\mathrm{Coefficients}\;\left[a_{1},a_{2},\ldots ,a_{p}\right]$			
	1-Order	2-Order	3-Order	4-Order
MITDB	[1]	[2, −1]	[3, −3, 1]	[4, −6, 4, −1]
PTBDB				
EDB				

**Table 5:** Average Determined Linear Prediction Coefficients.

Data Sets	$\mathrm{Average}\;\mathrm{Determined}\;\mathrm{Coefficients}\;\left[a_{1},a_{2},\ldots ,a_{p}\right]$			
	1-Order	2-Order	3-Order	4-Order
MITDB	[0.99]	[1.85, −0.86]	[2.18, −1.57, −0.38]	[2.10, −1.25, −0.06, 0.20]
PTBDB	[0.99]	[1.59, −0.60]	[1.54, −0.51, −0.04]	[1.53, −0.66, 0.40, −0.28]
EDB	[0.99]	[1.74, −0.75]	[2.13, −1.63, 0.50]	[2.14, −1.63, 0.46, 0.02]

**Table 6:** Rounded Average Determined Linear Prediction Coefficients.

Data Sets	$\mathrm{Rounded}\;\mathrm{Average}\;\mathrm{Determined}\;\mathrm{Coefficient}\;\left[a_{1},a_{2},\ldots ,a_{p}\right]$			
	1-Order	2-Order	3-Order	4-Order
MITDB	[1]	[2, −1]	[2, −2, 0]	[2, −1, 0, 0]
PTBDB	[1]	[2, −1]	[2, −1, 0]	[2, −1, 0, 0]
EDB	[1]	[2, −1]	[2, −2, 0]	[2, −2, 0, 0]

## Data Availability

The data presented in this study are openly available on Physio.net. PTB Diagnostic database at https://doi.org/10.13026/C28C71 (accessed on 24 January 2022), European ST-T database at https://doi.org/10.13026/C2D59Z (accessed on 24 January 2022), and MIT-BIH Arrhythmia database at https://doi.org/10.13026/C2F305 (accessed on 24 January 2022).
